# Positive feedback loop of hepatoma-derived growth factor and β-catenin promotes carcinogenesis of colorectal cancer

**DOI:** 10.18632/oncotarget.4982

**Published:** 2015-07-22

**Authors:** Jiayan Lian, Jianming Tang, Huijuan Shi, Hui Li, Tiantian Zhen, Wenlin Xie, Fenfen Zhang, Yang Yang, Anjia Han

**Affiliations:** ^1^ Department of Pathology, the First Affiliated Hospital, Sun Yat-Sen University, Guangzhou, China

**Keywords:** hepatoma-derived growth factor, β-catenin, colorectal cancer

## Abstract

To clarify the role of hepatoma-derived growth factor (HDGF) and β-catenin in carcinogenesis of colorectal cancer (CRC), our results showed that high HDGF expression was found in CRC cells and tissues and significantly related to histological differentiation (*p* = 0.035) and lymph node metastasis (*p* = 0.000). Significant positive correlation between HDGF expression and β-catenin abnormal expression was found in CRC tissues. High HDGF and lymph node metastasis were the strong independent prognostic indicators for reduced overall survival in CRC patients. HDGF knockdown dramatically inhibited cellular proliferation, migration, invasion, and tumorigenesis, both *in vitro* and *in vivo*, but induced G1 phase arrest and apoptosis in CRC cells. HDGF knock-down dramatically suppressed β-catenin and its down-stream genes expression in CRC cells. Intriguingly, β-catenin knock-down dramatically suppressed HDGF expression in CRC cells. Human recombinant Wnt3a and DKK1 treatment increased and decreased HDGF, β-catenin, c-Myc, cyclin D1, MMP9, and phos-GSK-3β (Ser9) protein expression in nuclear and cytoplasmic fraction of CRC cells upon β-catenin knock-down, respectively. Three HDGF-binding elements in β-catenin promoter were found and specific for transcriptional activation of β-catenin in CRC cells. In conclusion, our results first suggest that HDGF and β-catenin interacts as a positive feedback loop, which plays an important role in carcinogenesis and progression of CRC.

## INTRODUCTION

Colorectal cancer (CRC) is one of the most common malignancies worldwide. Hepatoma-derived growth factor (HDGF) is a heparin-binding protein that was originally purified from the conditioned media of human hepatocellular carcinoma cell line HuH-7, which proliferate autonomously in serum-free chemically defined medium [1]. HDGF is a prognostic marker in several types of cancer including hepatocellular carcinoma, esophageal squamous cell carcinoma, gastric cancer, intrahepatic cholangiocarcinoma, early-stage cervical adenocarcinoma, gallbladder cancer, and non small cell lung cancer [2-7]. Our recent study shows that HDGF exhibits oncogenic properties and may be a novel prognostic factor in Ewing's sarcoma [8]. HDGF expression is overexpressed in CRC and gradually increased in the colorectal carcinogenesis process [9, 10]. Blocking HDGF exhibits potent pro-apoptotic properties in CRC cells [10]. However, the molecular mechanism of HDGF involving in carcinogenesis of CRC remains unknown.

β-catenin is a key player in Wnt-signaling pathway. Oncogenic activation of Wnt- signaling pathway is mandatory for the initial neoplastic transformation of intestinal epithelium [11]. Our recent study shows that metastasis-associated in colon cancer 1 promotes carcinogenesis of CRC via β-catenin signaling pathway [12]. Astrocyte elevated gene-1 interacts with β-catenin and increases migration and invasion of CRC [13]. Zhang et al. recently report that Slit2/Robo1 signaling promotes intestinal tumorigenesis through Src-mediated activation of the Wnt/β-catenin pathway [14]. Anoikis of colon carcinoma cells triggered by beta-catenin loss can be enhanced by tumor necrosis factor receptor 1 antagonists [15]. Whether HDGF regulates β-catenin signaling pathway in CRC remains unknown. Our current study is to investigate whether HDGF regulates β-catenin signaling pathway in CRC and the underling mechanism.

## RESULTS

### HDGF and β-catenin expression in CRC cell lines and fresh tissues

**Figure 1:**
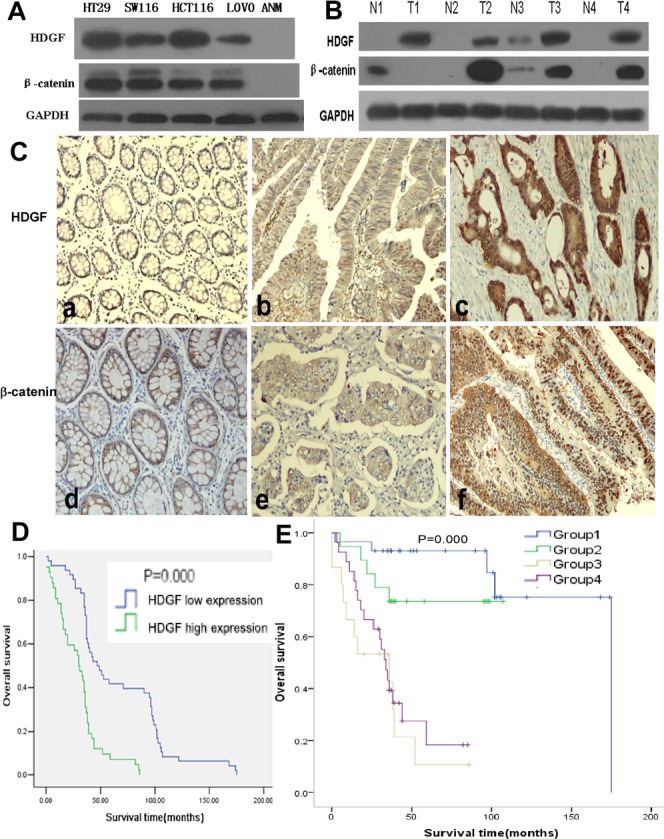
**A.**-**B.** HDGF and β-catenin protein expression in CRC cell lines (HT29, SW116, HCT116, and LOVO), non-tumor colorectal mucosa (ANM) tissues, 4 pairs of fresh CRC (T) and adjacent non-tumor colorectal mucosa (N) tissues by Western blot analysis; **C.** HDGF and β-catenin expression in CRC and ANM (a, d) by immunohistochemistry staining, low (a×100, b×200) and high (c×400) HDGF expression, normal (d×200, e×400) and abnormal (f×200) β-catenin expression; **D.** Overall survival (OS) of CRC patients with different levels of HDGF expression by Kaplan-Meier analysis; **E.** OS of CRC patients according to the combination of HDGF and β-catenin expression levels by Log-Rank test. Group 1: low HDGF/normal β-catenin expression; Group 2: low HDGF/abnormal β-catenin expression; Group3: high HDGF/normal β-catenin expression; Group 4: high HDGF/abnormal β-catenin expression.

HDGF and β-catenin protein expressions were higher in CRC cell lines including HT29, SW116, HCT116, and LOVO compared with non-tumor colorectal mucosa (ANM) by Western blot analysis, respectively. In addition, HDGF and β-catenin expression were dramatically increased in four samples of fresh CRC tissues compared with their respective ANM tissues by Western blot analysis, respectively (Figure 1A-1B).

### HDGF and β-catenin expression in paraffin-embedded CRC tissues and their relationship with clinicopathological features of CRC

In our series, HDGF positive signals were mostly located in the nuclei of CRC cells with minor cytoplasmic distribution by immunohistochemistry staining. Of 90 samples of paraffin-embedded CRC tissues, 42 samples (46.7%) were HDGF high expression, 48 samples (53.3%) were HDGF low expression. However, 14 samples (15.6%) were HDGF high expression, 76 samples (84.4%) were HDGF low expression in ANM tissue. HDGF high expression was significantly higher in CRC than that in their respective ANM tissues (*p* < 0.0001). HDGF expression was significantly related to histological differentiation (*p* = 0.035) and lymph node metastasis (*p* = 0.000). However, no significant relationship between HDGF expression and patients' age, gender, clinical stage, and tumor size was found (Table 1). In our series, 46 samples (51.1%) were β-catenin abnormal expression, 44 samples (48.9%) were β-catenin normal expression in CRC tissues (Figure 1C). However, 18 samples (20.0%) were β-catenin abnormal expression, 72 samples (80.0%) were β-catenin normal expression in ANM tissues. β-catenin abnormal expression was significantly higher in CRC than that in ANM tissues (*p* < 0.0001). There was a significantly positive correlation between HDGF expression and β-catenin abnormal expression in CRC tissues (r^2^ = 0.38, *p* < 0.001; Table 2).

**Table 1:** The relationship between HDGF expression and the clinicopathological features of CRC

Characteristics		HDGF expression			Overall survival analysis		
		Low (%)	High (%)	*P* value	5-year overall survival %	Univerate *P* value	Multivariate *P* value
Gender	Male	30(33.3)	19(21.1)	0.098	13.3		
	Female	18(20.0)	23(25.6)		12.2		
Age	≥60	23(25.6)	20(22.2)	0.101	15.5		
	<60	25(27.8)	22(24.4)		10.0		
Histological differentiation	Well	16(17.8)	14(15.6)	0.035	14.4	0.000	0.000
	Moderately	21(23.3)	9(10.0)		7.8		
	Poorly	11(12.2)	19(21.1)		3.3		
Clinical stage (Dukes)	A	4(4.4)	5(5.6)	0.367	11.1	0.003	0.049
	B	29(32.2)	18(20.0)		7.8		
	C	11(12.2)	12(13.3)		4.4		
	D	4(4.4)	7(7.8)		2.2		
Lymph node metastasis	Yes	16(17.8)	34(37.8)	0.000	3.3	0.000	0.000
	No	31(34.4)	9(10.0)		22.2		
The greatest diameter of tumor	≥5cm	9(10.0)	15(16.7)	0.069	7.7		
	<5cm	39(43.4)	27(30.0)		17.8		
HDGF expression	Low	48(53.3)			22.2	0.000	0.000
	High		42(46.7)		3.3		
HDGF/β-catenin expression	Group1				15.5	0.000	*
	Group2				7.8		
	Group3				6.6		
	Group4				2.2		

Group 1, Low HDGF/normal β-catenin expression; Group 2, Low HDGF/abnormal β-catenin; Group 3, high HDGF/normal β-catenin expression; Group 4, High HDGF/abnormal β-catenin expression. *HDGF/β-catenin expression was not included in multivariable Cox analysis in this Table.

**Table 2:** The correlation between HDGF expression β-catenin expression in CRC tissue

Variable		HDGF expression		*P* value	r^2^
		Low (%)	High (%)		
β-catenin expression	Normal	32 (35.6)	12(13.3)	<0.001	0.38
	Abnormal	16 (17.8)	30 (33.3)		

### Prognostic significance of HDGF and β-catenin expression in CRC

Kaplan-Meier analysis showed that CRC patients had a significantly lower overall survival (OS) rate in HDGF high expression group compared with that in HDGF low expression group (*p* = 0.000, Figure 1D). The OS rate for patients with HDGF high expression at 5 years was 3.3% compared with 22.2% for patients with HDGF low expression at 5 years. To determine whether HDGF expression was an independent prognostic factor for CRC patients, univariate Cox regression analysis indicated that HDGF high expression was significantly associated with reduced OS in CRC patients. In addition, other clinical parameters including histological differentiation, clinical stage, lymph node metastasis, and HDGF/β-catenin expression were also significant prognostic indicators for OS in CRC patients. Furthermore, multivariate Cox regression analysis demonstrated that HDGF high expression was an independent prognostic indicator for reduced OS in CRC patients. In addition, histological differentiation, clinical stage, and lymph node metastasis were independently associated with OS in CRC patients (Table 1).

We stratified our cohort of CRC patients into four groups according to the combination of different HDGF and β-catenin expression levels: group 1, low HDGF/normal β-catenin expression; group 2, low HDGF/abnormal β-catenin expression; Group 3, high HDGF/normal β-catenin expression and; and group 4, high HDGF/abnormal β-catenin expression. The 5-year OS rate (15.5%) of patients in group 1 was significantly higher than 6.6% for patients in group 3 (*p* = 0.000) and 2.2% for patients in group 4 (*p* = 0.000). Furthermore, the 5-year OS rate (7.8%) of patients in group 2 was significantly higher than 6.6% for patients in group 3 (*p* = 0.007) and 2.2% for patients in group 4 (*p* = 0.015). (Figure 1E). However, there was no significant difference of 5-year OS rate between group 1 and group 2 (*p* = 0.168), group 3 and group 4 (*p* = 0.387). Univariate and multivariate Cox regression analysis showed that high HDGF expression and lymph node metastasis were the strong independent prognostic indicators for reduced OS in CRC patients (Table 3).

**Table 3:** Multivariate Cox regression analysis for the prognostic value of clinicopathological parameters, HDGF and β-catenin expression in CRC

Characteristics		Hazard ratio	95% CI	*P* value
Histological differentiation	Well	0.723	0.549-0.987	0.145
	Moderately			
	Poorly			
Clinical stage (Dukes)	A	2.576	1.425 -1.932	0.076
	B			
	C			
	D			
Lymph node metastasis	Yes	4.234	1.723 -4.234	0.024
	No			
HDGF expression	Low	7.191	1.207-42.859	0.030
	High			
HDGF/β-catenin expression	Group1	1.042	0.527-2.062	0.906
	Group2			
	Group3			
	Group4			

Group 1, Low HDGF/normal β-catenin expression; Group 2, Low HDGF/abnormal β-catenin; Group3, high HDGF/normal β-catenin expression; Group 4, High HDGF/abnormal β-catenin expression

### HDGF knock-down reduced CRC cell growth

**Figure 2:**
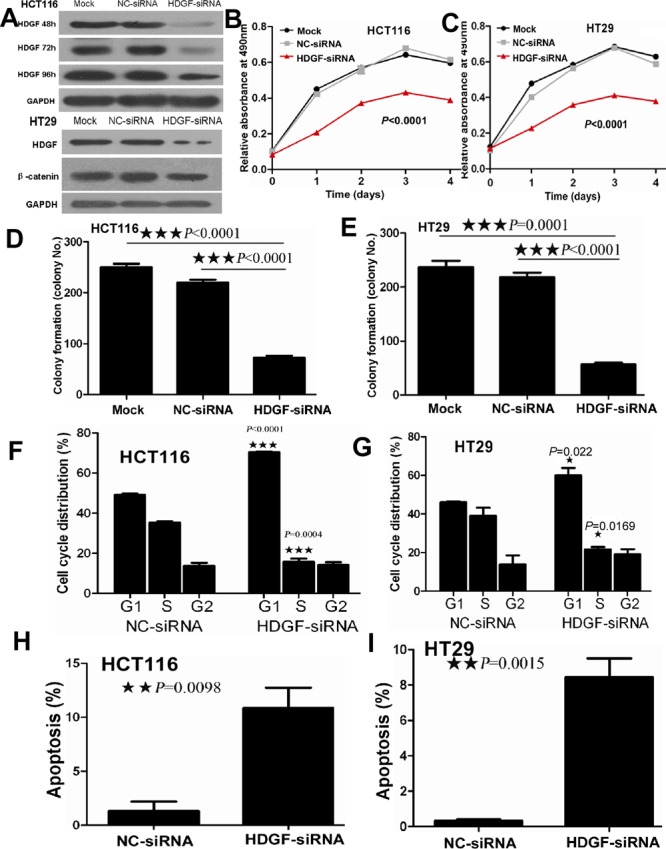
**A.** HDGF expression reduced in HCT116 cells transfected with HDGF-siRNA at 48, 72, and 96 hours and in HT29 cells transfected with HDGF-siRNA at 48 hours by Western blot analysis; **B.**-**C.** HDGF knockdown dramatically suppressed cell proliferation of HCT116 **B.** and HT29 **C.** cells compared with the control groups by MTS analysis, respectively; **D.**-**E.** HDGF knockdown significantly inhibited colony formation in HCT116 **D.** and HT29 **E.** cells compared with the control groups, respectively. The histogram showed the relative mean colony formation number; **F.**-**G.** HDGF knock-down resulted in G1 phase arrest in HCT116 **F.** and HT29 **G.** cells compared with NC-siRNA control group, respectively. **H.**-**I.** HDGF knock-down significantly induced cell apoptosis in HCT116 **H.** and HT29 **I.** cells compared with NC-siRNA control group, respectively.

As shown in Figure 2, HDGF protein level in HCT116 cells transfected with HDGF siRNA was significantly decreased compared with the control group and the effect lasted up to 96 hours after transfection. HDGF protein level in HT29 cells transfected with HDGF siRNA was significantly decreased compared with the control group. MTS assay showed that HDGF knockdown significantly suppressed HCT116 and HT29 cell proliferation compared with the NC-siRNA and Mock control groups at time dependent (p < 0.0001 and p < 0.0001), respectively. Furthermore, cell colony formation assay demonstrated that the mean number (mean number = 72) of colony formation in HCT116 transfected with HDGF-siRNA was significantly less than that in NC-siRNA transfection group (mean number = 229, *p* < 0.0001) and Mock control group (mean number = 251, *p* < 0.0001), respectively. Likewise, the mean number (mean number = 57) of colony formation in HT29 cells transfected with HDGF-siRNA was significantly less than that in NC-siRNA transfection group (mean number = 210, *p* < 0.0001) and Mock control group (mean number = 237, *p* < 0.0001), respectively. The cell colony formation rate was also significantly suppressed about 60% and 60% in HCT116 and HT29 cells transfected with HDGF-siRNA compared with NC-siRNA transfection group (*p* < 0.0001 and *p* < 0.0001) and Mock control group (*p* < 0.0001 and *p* = 0.0001), respectively.

### HDGF knock-down suppressed cell cycle progression and induced cell apoptosis in CRC cells

As shown in Figure 2F-2I, cell cycle analysis showed that HDGF knock-down significantly induced G1-S phase arrest in HCT116 (*p* < 0.0001) and HT29 (*p* = 0.022) compared with the control group, respectively. After 48 hours transfection, the proportion of late apoptotic HCT116 cells which were positive for Annexin-V and PI was 14.5% in HDGF siRNA transfection group compared with 1% of late apoptotic cells in the control group. The relative percentage of late apoptotic HCT116 cells increased by 14-fold of the control group (*p* = 0.0098). Likewise, late apoptotic cells (9.5%) of HT29 cells transfected with HDGF-siRNA were more than 0.464% of late apoptotic cells in the control group. The relative percentage of late apoptotic HT29 cells increased by 20-fold of the control group (*p* = 0.0015).

### HDGF knock-down inhibited migration and invasion of CRC cells

**Figure 3:**
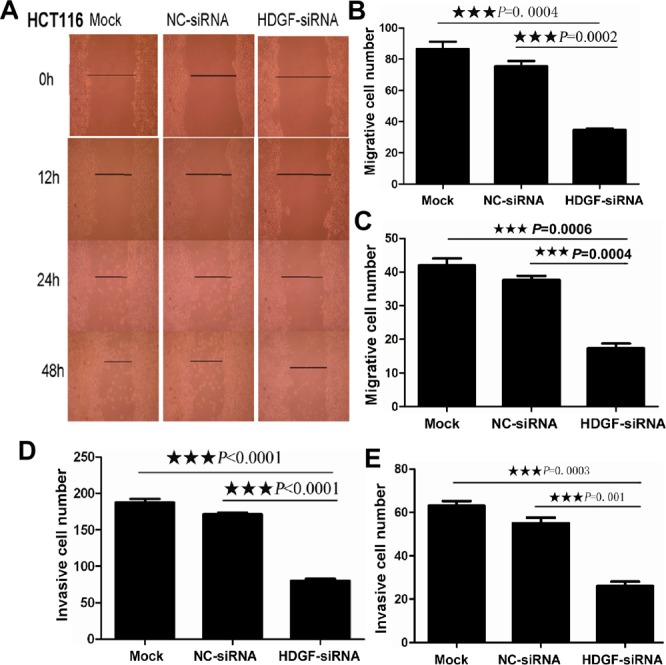
**A.** HDGF knockdown inhibited cell migration in HCT116 by scratch wound assay; **B.**-**E.** HDGF knockdown inhibited cell migration **B.**-**C.** and invasion **D.**-**E.** in HCT116 **B.**, **D.** and HT29 **C.**, **E.** by transwell migration and transwell invasion assay compared with the control groups, respectively.

As shown in Figure 3, scratch wound assay showed that cell migration was dramatically inhibited in HCT116 cells transfected with HDGF-siRNA compared with Mock and NC-siRNA-transfected control groups at 12, 24, and 48 hours, respectively. Furthermore, transwell migration assay showed that the mean number (mean number = 35) of migrated cells per field of view was significantly decreased in HCT116 transfected with HDGF-siRNA than that in NC-siRNA transfection group (mean number = 76, *p* = 0.0002) and Mock control group (mean number = 87, *p* = 0.0004), respectively. Likewise, the mean number (mean number = 17) of migrated cells per field of view was significantly decreased in HT29 transfected with HDGF-siRNA than that in NC-siRNA transfection group (mean number = 38, *p* = 0.0004) and Mock control group (mean number = 42, *p* = 0.0006), respectively. Transwell matrix penetration assay showed that the mean number (mean number = 80) of invasive cells was significantly fewer in HCT116 cells transfected with HDGF-siRNA group than that in NC-siRNA transfection group (mean number = 162, *p* < 0.0001) and Mock control group (mean number = 195, *p* < 0.0001), respectively. Likewise, the mean number (mean number = 26) of invasive cells was significantly fewer in HT29 cells transfected with HDGF-siRNA group than that in NC-siRNA transfection group (mean number = 55, *p* = 0.001) and Mock control group (mean number = 63, *p* = 0.0003), respectively.

### HDGF knock-down inhibited the tumorigenicity of CRC cells *in vivo*

**Figure 4:**
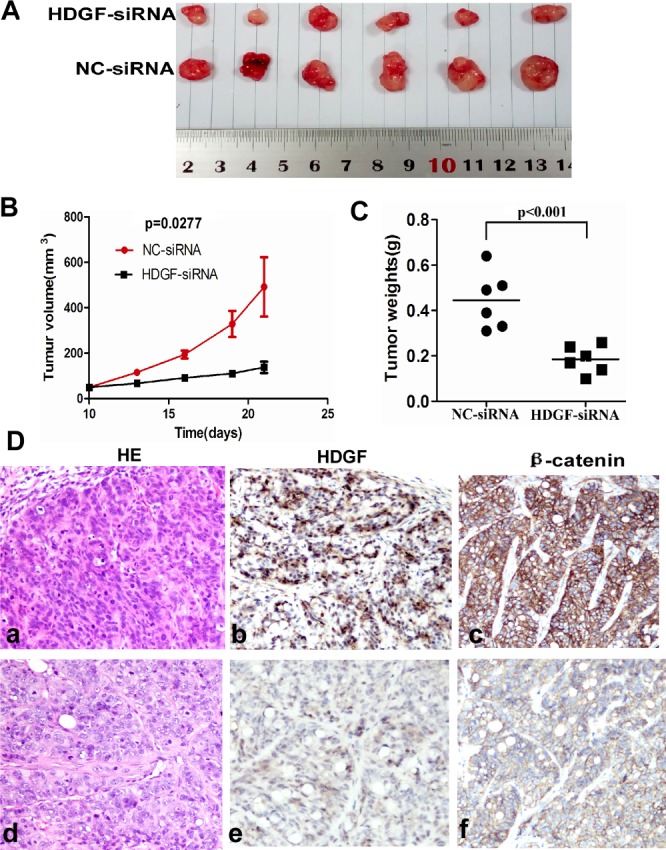
**A.**-**C.** HDGF knock-down significantly suppressed tumor growth **A.**-**B.** and reduced tumor weight **C.** of HT29 cells implanted subcutaneously in BALB/c-nu mice compared with the control group, respectively; **D.** Histological features of tumor xenograft from NC-siRNA group (a**).** and HDGF-siRNA group (**d).** by haematoxylin and eosin staining; lower HDGF and β-catenin expression were found in tumors injected with HDGF-siRNA (e, f) compared with NC-siRNA control group (b, c) by immunohistochemistry staining ×400, respectively.

To determine whether HDGF affects the tumorigenicity of CRC cells *in vivo*, we performed tumor growth experiments in nude mice using HT29 cells. As shown in Figure 4A-4B, tumor growth was significantly inhibited in HDGF-siRNA treatment group compared with the control group (*p* = 0.0277). The control group exhibited a rapid increase in tumor volume over 12 days. However, HDGF siRNA treatment group showed a low increase in tumor volume. Concomitantly, the average final tumour weight in the treatment group was significantly less than that in control group upon termination of the experiment (*p* < 0.001, Figure 4C). Moreover, histological features of tumor xenograft from NC-siRNA group and HDGF-siRNA group were similar to human CRC tissues by haematoxylin and eosin staining. However, we detected HDGF and β-catenin expression in enucleated tumors by immunohistochemistry staining. The results showed that tumors treated with HDGF-siRNA displayed dramatically low HDGF and β-catenin expression compared with NC-siRNA control group, respectively (Figure 4D).

### Exogenous HDGF enhanced cell proliferation in CRC cells

**Figure 5:**
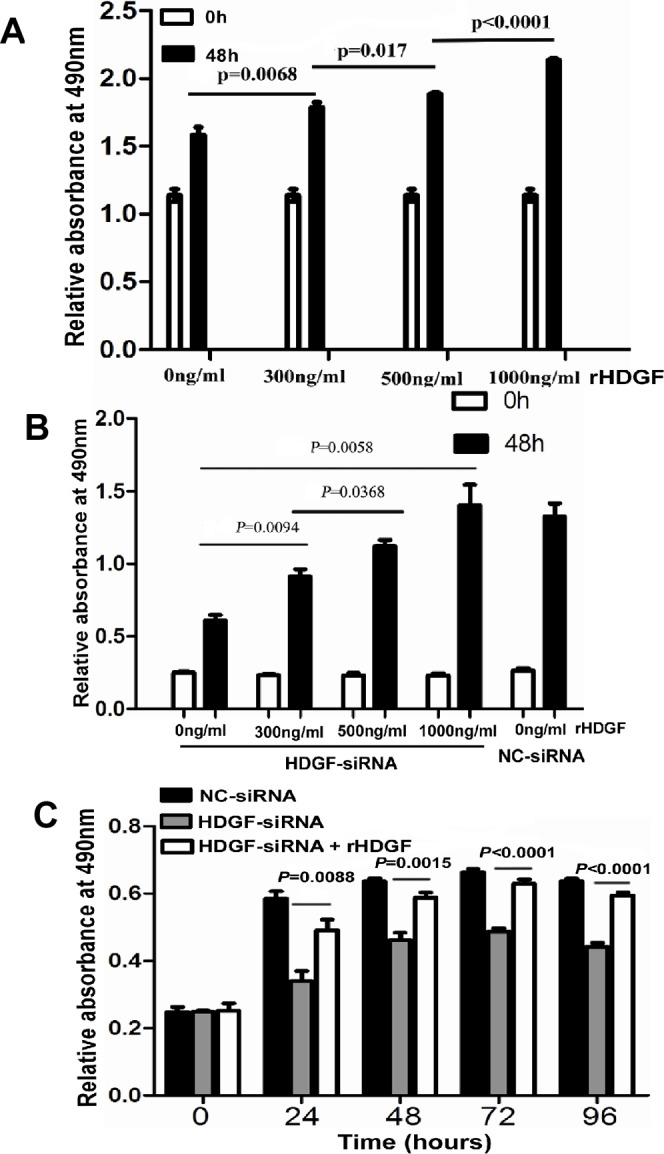
**A.**-**C.** Recombinant HDGF (rHDGF) significantly increased cell proliferation of LOVO cells **A.** and reversed cell proliferation suppression of HCT116 upon HDGF knock-down in a dose-dependent manner **B.** and time-dependent manner **C.** by MTS assay, respectively.

It was interesting that HDGF protein expression was low in LOVO cells and was high in HCT116 cells by Western blot analysis. Therefore, LOVO cell was a good *in vitro* model for us to study the biological role of exogenous HDGF in CRC cell. First, LOVO cells were serum-starved for synchronization. Quiescent cells were then treated with four different concentrations (0ng/ml, 300ng/ml, 500ng/ml, 1000ng/ml) of human recombinant HDGF (rHDGF) for 48 hours and cell growth was measured by MTS assay. As shown in Figure 5A, rHDGF promoted LOVO cell growth in dose-dependent manner. The lower concentration of rHDGF (300ng/ml) increased LOVO cell growth by 18% compared with the untreated control group (*p* = 0.0068). 500ng/ml rHDGF further enhanced cell growth by 12% in comparison with cells treated with 300ng/ml rHDGF (*p* = 0.017). The higher concentration (1000ng/ml) of rHDGF dramatically promoted LOVO cell growth by 21% in comparison with cells treated with 500ng/ml rHDGF (*p* < 0.0001).

The rescue assay was further performed in HCT116 cells. The suppression of cell growth in HCT116 with HDGF knock-down was dramatically attenuated in dose-dependent manner by rHDGF treatment. At a dose of 300ng/ml rHDGF treatment enhanced the cell growth by 30% of the untreated control cells (*p* = 0.0094). 500ng/ml rHDGF treatment further enhanced cell growth by 30% in comparison with cells treated with 300ng/ml rHDGF (*p* = 0.0368). 1000ng/ml rHDGF treatment further enhanced cell growth by 100% compared with the untreated control cells (*p* = 0.0058). In particular, 1000ng/ml rHDGF reversed the cell growth suppression by HDGF knock-down near to the level of HCT116 cells transfected with NC-siRNA (Figure 5B). 500ng/ml rHDGF significantly rescued the cell growth suppression of HCT116 with HDGF knock-down at 24, 48, and 72 hours (*p* = 0.0088, *p* = 0.0015, and *p* < 0.0001), respectively and the effect even lasted up to 96 hours (*p* < 0.0001) (Figure 5C).

### HDGF knockdown inhibited β-catenin signaling in CRC cells

**Figure 6:**
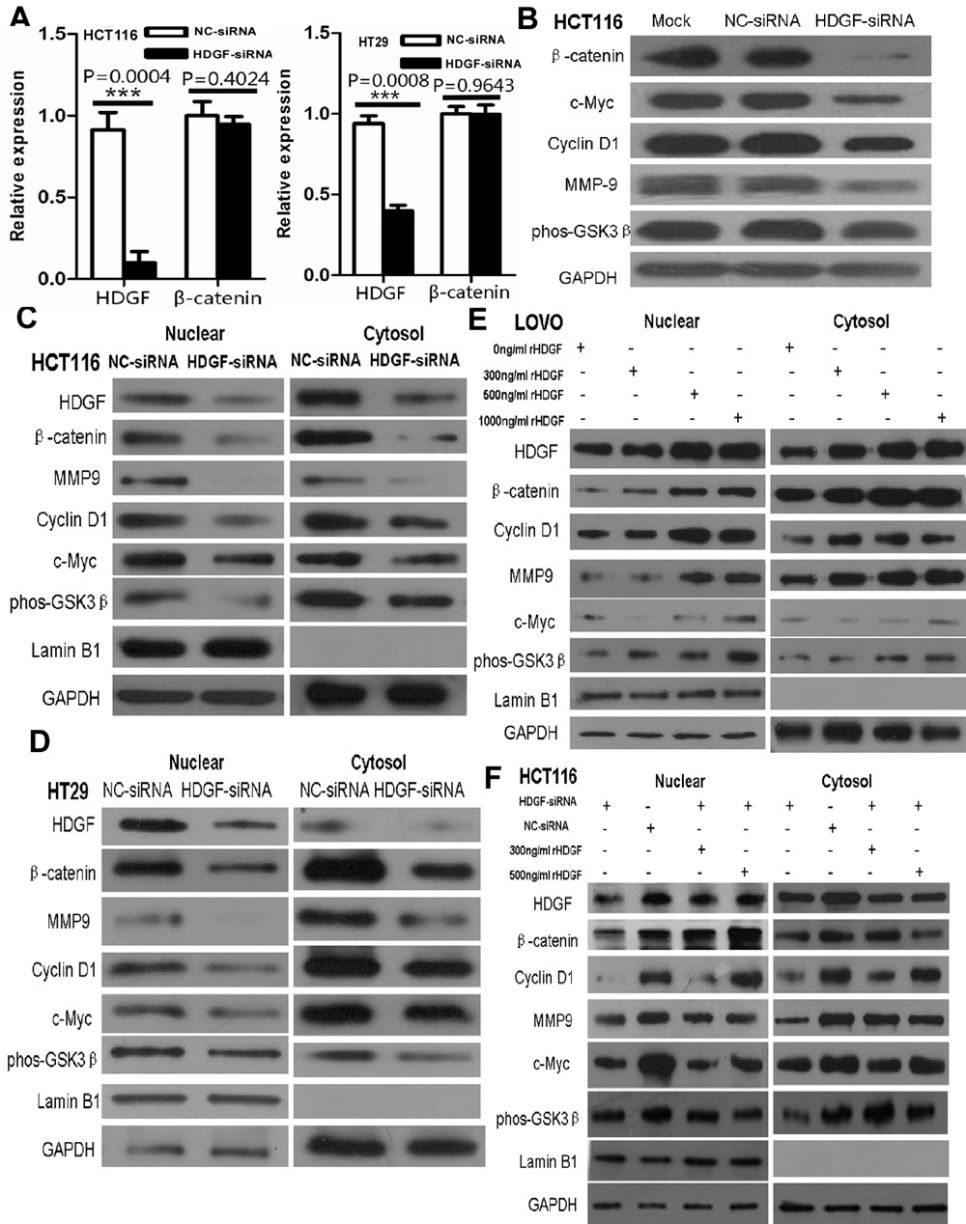
**A.** β-catenin mRNA expression was not significantly altered in HCT116 and HT29 cells with HDGF knockdown by real-time PCR analysis, respectively; **B.** HDGF knockdown dramatically inhibited β-catenin and c-Myc, cyclin D1 and MMP9 and phos-GSK3β (Ser9) expression in HCT116 cells; **C.**-**D.** Nuclear and cytoplasmic β-catenin, c-Myc, cyclin D1, MMP9, and phos-GSK-3β (Ser9) expression were suppressed in HCT116 **C.** and HT29 **D.** cells with HDGF knock-down; **E.** rHDGF enhanced nuclear and cytoplasmic β-catenin, c-Myc, cyclin D1, MMP9, and phos-GSK-3β(Ser9) protein expression in LOVO cells; **F.** rHDGF reversed the nuclear and cytoplasmic protein expression suppression of β-catenin c-Myc, cyclin D1, MMP9, and phos-GSK-3β (Ser9) in HCT116 upon HDGF knock-down by Western blot analysis. GAPDH was considered as loading control. Lamin B1 was considered as nuclear loading control.

To determine whether HDGF affects β-catenin transcriptional level in CRC cells, our data showed that β-catenin mRNA expression was not significantly altered in HCT116 and HT29 cells with HDGF knockdown by real-time PCR analysis (Figure 6A). However, HDGF knockdown dramatically inhibited β-catenin and its down-stream genes including c-Myc, cyclin D1 and MMP9 and up-stream gene phos-GSK3β (Ser9) protein expression in HCT116 compared with NC-siRNA and Mock control groups by Western blot analysis (Figure 6B). Likewise, HDGF knockdown dramatically inhibited β-catenin protein expression in HT29 cells (Figure 2A). Further study showed that decreased nuclear and cytoplasmic β-catenin and its down-stream genes including c-Myc, cyclin D1 and MMP9 and up-stream gene phos-GSK3β (Ser9) expression level were found in HCT116 and HT29 cells with HDGF-siRNA compared with the control group, respectively (Figure 6C-6D).

To study the effect of exogenous HDGF on β-catenin signaling of CRC cells, nuclear and cytoplasmic HDGF, β-catenin, c-Myc, cyclin D1, MMP9 and phos-GSK-3β (Ser9) expression increased in LOVO cells in dose dependent manner. Furthermore, β-catenin, c-Myc, cyclin D1, MMP9 and phos-GSK-3β (Ser9) expression suppression was significantly attenuated in HCT116 with HDGF knock-down by 300ng/ml and 500ng/ml rHDGF treatment compared with the untreated group, respectively (Figure 6E-6F).

### β-catenin knock-down suppressed HDGF expression in CRC lines

**Figure 7:**
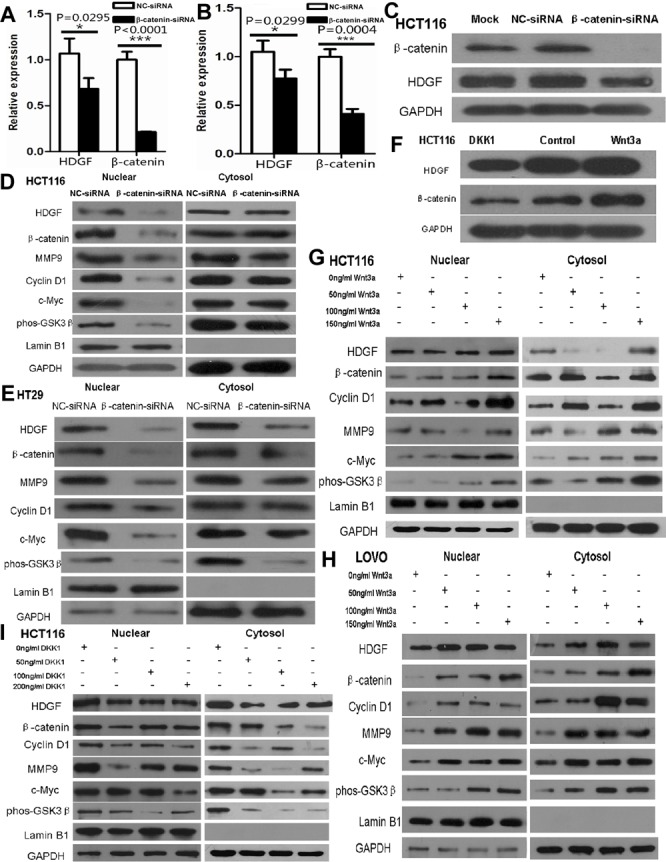
**A.**-**B.** β-catenin knockdown significantly suppressed HDGF mRNA expression in HCT116 **A.** and HT29 **B.** cells by real-time PCR analysis, respectively; **C.**-**E.** β-catenin knockdown inhibited HDGF protein expression in HCT116 cells **C.** and mainly inhibited nuclear HDGF, β-catenin, c-Myc, cyclin D1, MMP9 and phos-GSK-3β (Ser9) protein expression in HCT116 **D.** and HT29 **E.** cells; **F.** Recombinant Wnt3a and DKK1 increased and decreased HDGF and β-catenin expression in HCT116 cells, respectively; **G.**-**I.** Recombinant Wnt3a and DKK1 increased and decreased nuclear and cytoplasmic HDGF, β-catenin, c-Myc, cyclin D1, MMP9 and phos-GSK-3β (Ser9) protein expression in HCT116 **G.**, **I.** and LOVO **H.** cells by Western blot analysis, respectively.

As shown in Figure 7A-7B, β-catenin knockdown significantly inhibited HDGF mRNA expression in HCT116 and HT29 cells by real-time PCR analysis. HDGF and β-catenin protein expression were dramatically suppressed in HCT116 cells transfected with β-catenin siRNA (Figure 7C). Furthermore, nuclear HDGF, β-catenin, c-Myc, cyclin D1, MMP9 and phos-GSK3β (Ser9) expression were reduced in HCT116 and HT29 cells compared with the control group at 48 hours by Western blot analysis, respectively (Figure 7D-7E).

It is well known that human recombinant Wnt-3a and DKK1 can agonist and inhibit Wnt/β-catenin pathway, respectively. To further verify the effect of β-catenin on HDGF expression in CRC cells, HDGF and β-catenin protein expressions in HCT116 were induced by 100ng/ml human recombinant Wnt3a (R&D SYSTEMS) and inhibited by 200ng/ml human recombinant DKK1 (R&D SYSTEMS) for 48 hours by Western blot analysis, respectively (Figure 7F). Furthermore, as shown in Figure 7G-7H, nuclear and cytoplasmic HDGF, β-catenin, c-Myc, cyclin D1, MMP9, and phos-GSK-3β (Ser9) protein expression increased in HCT116 and LOVO cells treated with Wnt3a at a concentration of 50ng/ml, 100ng/ml, and 150ng/ml for 48 hours compared with the control group, respectively. Nuclear and cytoplasmic HDGF, β-catenin, c-Myc, cyclin D1, MMP9, and phos-GSK-3β (Ser9) protein expression decreased in HCT116 cells treated with DKK1 at 50ng/ml, 100ng/ml, and 200ng/ml for 48 hours compared with the control group, respectively (Figure 7I).

### Positive feedback loop of HDGF and β-catenin in CRC cells

**Figure 8:**
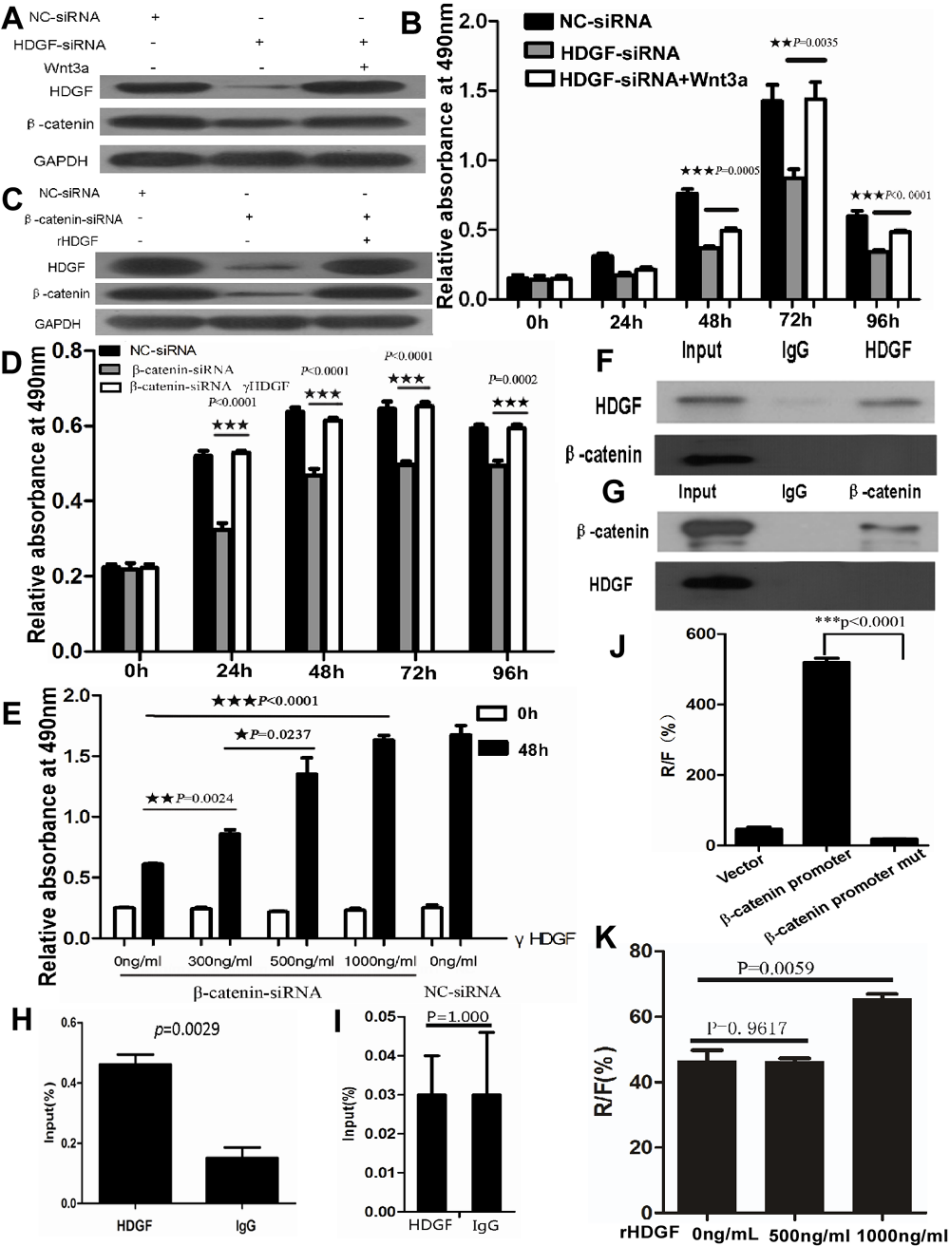
**A.**-**B.** Recombinant Wnt3a reversed HDGF and β-catenin protein expression suppression **A.** and cell proliferation suppression **B.** in HCT116 cells with HDGF knock-down by Western blot analysis and MTS assay, respectively; **C.**-**E.** rHDGF reversed HDGF and β-catenin protein expression suppression **C.** and cell proliferation suppression **D.**-**E.** in HCT116 cells with β-catenin knock-down by Western blot analysis and MTS assay in time-dependent manner **D.** and dose-dependent manner **E.**, respectively. **F.**-**G.** Direct interaction of HDGF protein and β-catenin protein was not found in HCT116 cells by co-immunoprecipitation assay; **H.** The region of β-catenin promoter from immunoprecipitated chromatin DNA in HCT116 cells using HDGF antibody was amplified by ChIP and real-time PCR analysis. **I.** The region of β-catenin promoter from immunoprecipitated chromatin DNA was not amplified in HCT116 with HDGF knockdown compared with the control group; **J.** β-catenin transcriptional activity significantly decreased in HCT116 cells transfected with the mutant β-catenin promoter luciferase reporter compared with the wild type of β-catenin promoter group by dual-luciferase reporter assay; **K.**1000ng/ml rHDGF significantly increased β-catenin transcriptional activity in HCT116 cells compared with the control group by dual-luciferase reporter assay (*p* = 0.0059).

To further study whether there is a positive feedback loop of HDGF and β-catenin in CRC cells. HDGF knock-down resulted in HDGF and β-catenin expression suppression in HCT116 cells by Western blot analysis. Interestingly, HDGF and β-catenin expression suppression was dramatically reversed by 100ng/ml Wnt3a treatment for 48 hours. Likewise, β-catenin knock-down resulted in β-catenin and HDGF expression suppression in HCT116 cells by Western blot analysis. However, β-catenin and HDGF expression suppression was dramatically reversed by 500ng/ml rHDGF treatment for 48 hours (Figure 8).

To further determine the effect of HDGF and β-catenin positive feedback loop on cell proliferation, as shown in Figure 8B, 100ng/ml Wnt3a treatment dramatically reversed cell proliferation suppression of HCT116 with HDGF knock-down at 48, 72, and 96 hours compared with HDGF-siRNA group without Wnt3a treatment, respectively by MTS analysis. Likewise, 500ng/ml rHDGF dramatically reversed cell proliferation suppression of HCT116 transfected with β-catenin siRNA at 24, 48, 72, and 96 hours compared with β-catenin siRNA group without rHDGF, respectively. Moreover, the cell proliferation suppression of HCT116 cells transfected with β-catenin siRNA was dramatically attenuated by rHDGF treatment in dose-dependent manner. At a dose of 300ng/ml, rHDGF enhanced the cell growth by 20% of the untreated control cells (*p* = 0.0024). 500ng/ml rHDGF enhanced cell growth by 50% in comparison with cells treated with 300ng/ml rHDGF (*p* = 0.0237). 1000ng/ml rHDGF further enhanced cell growth by 150% of the untreated cells (*p* < 0.0001) (Figure 8D-8E).

### The mechanism of positive feedback loop of HDGF and β-catenin in CRC cells

To study whether HDGF protein directly interacts with β-catenin protein in CRC, our result showed that no direct interaction of HDGF protein and β-catenin protein was found in HCT116 cells by co-immunoprecipitation assay (Figure 8F-8G). However, we found that the human β-catenin promoter region contained putative HDGF-binding elements in three regions by sequence alignment analysis using JASPAR database. Antibody-specific ChIP assay was used to determine whether these three regions (-54 to -47bp, -157 to -150bp, and -648 to -640bp) of β-catenin promoter region could bind the endogenous HDGF protein in HCT116 cells, the primers for real time-PCR covering the region from -484 to -288bp were used. We successfully amplified this specific region (-484 to -288bp) from immunoprecipitated chromatin DNA by ChIP and real-time PCR using HDGF antibody in HCT116 cells compared with the control group (*p* = 0.0029, Figure 8H). However, this specific region in HCT116 transfected with HDGF-siRNA at 48 hours was not amplified compared with the control group (*p* = 1.000, Figure 8I). To further determine the specificity of HDGF-binding elements in β-catenin promoter region, we performed site-specific mutagenesis within the three putative HDGF-binding regions of β-catenin promoter. Dual-luciferase reporter assay showed that β-catenin transcriptional activity significantly decreased in HCT116 cells transfected with the mutant β-catenin promoter luciferase reporter compared with the wild type of β-catenin promoter (*p* < 0.0001, Figure 8J). Moreover, 500ng/ml rHDGF did not significantly affect β-catenin transcriptional activity in HCT116 cells compared with the control group (*p* = 0.9617), but 1000ng/ml rHDGF significantly increased β-catenin transcriptional activity in HCT116 cells compared with the control group by dual-luciferase reported assay (*p* = 0.0059, Figure 8K).

## DISCUSSION

The dramatically negative impact of HDGF on survival has been reported in various cancers [16, 17]. Liao et al. reported that positive HDGF staining correlated with lymph node metastasis, the degree of differentiation, and Dukes stage in CRC [10]. Our data showed that HDGF expression was significantly related to histological differentiation and lymph node metastasis in CRC. Multivariate Cox regression analysis demonstrated that high HDGF expression and lymph node metastasis were independent prognostic indicators for reduced OS in CRC patients. The results indicate that HDGF might be a novel biomarker for CRC.

Recent study shows that blocking HDGF exhibits potent pro-apoptotic properties in CRC cells [10]. To fully clarify the biological significance of HDGF in CRC, our data demonstrated that HDGF knockdown dramatically inhibited cellular proliferation, migration, invasion, colony formation, and tumorigenesis, both *in vitro* and *in vivo*, but induced G1 phase arrest and apoptosis in CRC cells. Furthermore, human recombinant HDGF enhanced CRC cell growth and partially retrieved the cell growth suppression in CRC cells upon HDGF knock-down. Ren et al. reported anti-HDGF monoclonal antibodies, alone or in combination with bevacizumab/avastin and/or gemcitabine, could effectively inhibit tumour growth in non-small cell lung cancer xenograft models [18]. Liao et al. reported over-expression of HDGF could confer the resistance of human CRC cells to nordihydroguaiaretic acid toxicity [19]. Whether CRC patients could dramatically benefit from anti-HDGF antibodies, alone or in combination with other chemotherapeutic agents, needs further study.

The molecular mechanism of HDGF in CRC remains unknown. Mao et al. have reported that HDGF is involved in the gastric carcinogenesis process and promotes proliferation and metastasis via Erk1/2 activation [20]. Song et al. have reported that HDGF regulates glioma cell growth, apoptosis and epithelial-mesenchymal transition probably through the Akt and the TGF-beta signaling pathways [21].

Mutations in adenomatous polyposis coli (APC) or β-catenin result in the accumulation and nuclear translocation of β-catenin and in β-catenin/ T-cell factor (TCF) 4-regulated transcription of TCF target genes such as cyclin D1, c-Myc, and phopholipase D [22]. Our recent study has found that astrocyte elevated gene-1 interacts with β-catenin and increases migration and invasion of CRC [13]. Our data showed that there was a positive correlation between HDGF expression and β-catenin in CRC. HDGF knock-down dramatically suppressed β-catenin and its down-stream genes expression in CRC cells. However, rHDGF significantly attenuated nuclear and cytoplasmic HDGF, β-catenin and its down-stream genes expression in CRC cells upon HDGF knock-down. Intriguingly, β-catenin knock-down dramatically suppressed HDGF mRNA and protein expression in CRC cells. Wnt3a and DKK1 treatment increased and decreased HDGF, β-catenin, c-Myc, cyclin D1, MMP9, and phos-GSK-3β (Ser9) protein expression in nuclear and cytoplasmic fraction of CRC cells upon β-catenin knock-down, respectively. Our results first indicate that HDGF and β-catenin interact as a positive feedback loop in CRC and has an effect on CRC cell proliferation. Further study showed that there was no direct interaction of HDGF protein and β-catenin protein in HCT116 cells by co-immunoprecipitation assay. Lukasik et al. have reported a protein DNA -binding site in HDGF PWWP domain [23]. Yang and Everett have reported that HDGF binds DNA through the N-terminal PWWP domain [24]. Our data first showed that three HDGF-binding regions in β-catenin promoter were found and specific for transcriptional activation of β-catenin in HCT116 cells. 500ng/ml rHDGF did not affect β-catenin transcriptional activity in HCT116 cells. But rHDGF at a concentration of 1000ng/ml could significantly increase β-catenin transcriptional activity in HCT116 cells compared with the control group. The reason might be related to HCT116 cells containing an activating β-catenin mutation. This issue needs further study in other CRC cell lines. Based on the above data, our hypothesis is that HDGF binds the HDGF-binding regions in the promoter of β-catenin, which results in β-catenin transcriptional activation and its down-stream genes' expression; activated β-catenin further promotes HDGF expression, so the positive feedback loop of HDGF and β-catenin promotes the proliferation, migration, and invasion of CRC cells.

In conclusion, our data first suggest that HDGF and β-catenin interacts as a positive feedback loop, which plays an important role in carcinogenesis and progression of CRC.

## MATERIALS AND METHODS

### Cell lines and small interfering RNA (siRNA) sequences

The human CRC cell line HCT116 was maintained in McCoy's 5A medium (Invitrogen, Carlsbad, CA). LOVO and SW116 were cultured in RPMI-1640 medium (Invitrogen). HT29 was cultured in Dulbecco's modified Eagle's medium (Invitrogen). All medium was supplemented with 10% (v/v) fetal bovine serum (Invitrogen), 1×antibiotic/antimycotic (100 units/mL streptomycin, 100units/mL penicillin, and 0.25 mg/mL amphotericin B). All cell lines were cultured in humidified incubator at 37°C with 5% CO_2_.

The targeted HDGF sequences were: sense, 5′- CAA GGA GAA GAA CGA GAA AdTdT-3′. The targeted β-catenin sequences were: sense, 5′-GCC ACA AGA UUA CAA GAA AdTdT-3′. The siRNA duplexes were chemically synthesized and purified by Ribobio Co. Ltd (Guangzhou, China). The siRNA was transfected using Lipofectamine RNAiMAX transfection reagent (Invitrogen). Lipofectamine RNAiMAX alone (Mock) and scrambled siRNA (NC-siRNA) were used as negative control groups.

### Patient information and tissue specimens

A total of 90 samples of paraffin-embedded CRC tissues and four pairs of fresh CRC tissues and their respective ANM samples between 1990 and 2009 were collected from Department of Pathology, the first Affiliated Hospital, Sun Yat-sen University, Guangzhou, China. Prior patient consent and approval from the Institutional Research Ethics Committee were obtained. No patients had received chemotherapy or radiotherapy before operation. The histopathology of the disease was determined by two pathologists according to the criteria of the WHO. Clinical staging was done according to Dukes classification. Pertinent follow-up information was available for all patients. Detailed clinical information is summarized in Table 1.

### Cell proliferation assay

HCT116 and HT29 cells (1×10^3^) were plated onto 96-well plates with medium containing 10% FBS and incubated overnight. After transfection with 100nM HDGF siRNA, cell proliferation was determined at 0, 24, 48, 72, and 96 hours using CellTiter 96R Aqueous One Solution Cell Proliferation Assay (MTS) (Promega, Madison, WI). The absorbance (OD) was measured at a wavelength of 490 nm using a Microplate Autoreader (Bio-Tek Instruments, VT). Mock and cells transfected with NC-siRNA were used as negative control groups. This experiment was performed in triplicate.

### Colony formation assay

After 100nM HDGF siRNA transfection for 48 hours, a total of 400 HCT116 and HT29 cells were plated onto 6-well plates and incubated at 37^o^C in a 5% CO_2_ incubator for 2 weeks, respectively. Fresh medium was added every 3 days. At the end-point, the cells were washed with cold phosphate buffered saline (PBS) twice, fixed with 4% paraformaldehyde for 30 minutes and stained with 1% crystal violet solution for 20 minutes at room temperature. The visible colony numbers were counted. Mock and NC-siRNA were used as negative control groups. This experiment was performed in triplicate.

### Cell cycle and apoptosis assay

HCT116 and HT29 cells (3×10^5^) were seeded in 6-well plates and incubated overnight until 50%-60% confluent, then transfected with 100nM HDGF siRNA for 48 hours, washed in cold PBS, fixed with 70% cold ethanol for 24h at 4^o^C, then stained with Annexin V-FITC binding buffer and propidium iodide buffer (50mg/ml propidium iodide, 0.1% sodium citrate and 0.1% Triton X-100) for 30 minutes at room temperature. 2×10^4^ cells were analyzed for cell cycle and apoptosis using a Becton Dickinson FACScan (Becton Dickinson Immunocytometry Systems, San Jose, CA). The percentage of cells in each phase of the cell cycle and apoptotic cells was quantified using Cell Quest software, respectively. This experiment was performed in triplicate.

### Scratch wound assay

HCT116 cells were plated in 6-well plates and incubated overnight until 30%-50% confluent, then transfected with 100nM HDGF siRNA. Vertical scratches were then made using a 100μl plastic filter tip to create a ‘wound’ of approximately 100μm in diameter. To eliminate dislodged cells, culture medium was removed and wells were washed with PBS. ‘Wound closure’ was observed at 0, 12, 24, 48 hours and digital images were taken under an inverted microscope.

### Transwell migration and invasion assays

Migration and invasion assays were carried out in Transwell chambers containing polycarbonate filters (8μm pore size; Corning Incorporated, Life Sciences, NY). After transfected with 100nM HDGF siRNA for 48h, 2×10^3^ (migration assay) or 2×10^4^ (invasion assay) HCT116 cells and HT29 cells in a 500μl volume of serum-free medium were placed in the upper chambers and incubated at 37^o^C with 5% CO_2_ for 24 hours, respectively. While a 200μl volume of medium containing 15% FBS was added to the lower chamber as chemoattractant. Cells were allowed to invade through the matrigel (BD Biosciences) or migrate for 24 hours at 37^o^C with 5% CO_2_. Following invasion or migration, cells were fixed with 4% formaldehyde and stained with 1% crystal violet. Cells on the upper surface of the filters were removed by wiping with a cotton swab. Cells counts were the mean number of cells per fields of view. Three independent experiments were performed and the data were presented as mean±standard deviation (SD).

### Cellular fractionation

Cultured cells were collected and resuspended in 500μl 1×hypotonic buffer on ice. 25μl detergent was added and vortexed for 10 seconds at highest settings. Suspension was centrifuged for 30 seconds at 14000*g* in a microcentrifuge tube and precooled at 4^o^C. The supernatant (cytoplasmic fraction) was transferred into a prechilled microcentrifuge tube. Nuclear pellet was resuspended in 50μl complete lysis buffer and centrifuged for 10 minutes at 14000*g* in a microcentrifuge tube and precooled at 4^o^C. Supernatant (nuclear fraction) was transferred into a prechilled microcentrifuge tube.

### Quantitative real-time PCR analysis

Quantitative real-time PCR was run on ABI StepOne Plus PCR system (Applied Biosystems). The primer sequences used for HDGF were followed: forward: 5′-AGG CGG AAA CCG TGT A-3′; reverse: 5′-CCA GGA ATG CCG TCT C-3′. The primer sequences used for β-catenin: forward: 5′-TTG AAA ATC CAG CGT GGA CA-3′; reverse: 5′-TCG AGT CAT TGC ATA CTG TC-3′. The geometric mean of housekeeping gene glyceraldehyde-3-phosphate dehydrogenase (GAPDH) was used to normalize the variability at mRNA expression levels. All experiments were performed in triplicate.

### Western blot analysis

As described previously [8], 25 μg of total proteins were loaded onto 10% sodium dodecyl sulphate polyacrylamide gel electrophoresis (SDS-PAGE) and transferred onto polyvinylidene difluoride (PVDF) membranes (Millipore, Billerico, MA) that were subsequently blocked in 5% non-fat milk in TBST (20 mmol/L Tris, pH 7.6, 137 mmol/L NaCl, 0.1% Tween-20). The membranes were incubated with primary antibodies including rabbit HDGF (Abcam, dilution 1:1000), rabbit β-catenin, rabbit cyclin D1, rabbit c-Myc, rabbit phosphorylated- glycogen synthase kinase 3β (phos-GSK-3β) (Ser9), and rabbit MMP9 (Cell Signaling Technology, Danvers, MA, dilution 1:1000) at 4°C overnight. Rabbit anti-glyceraldehyde-3-Phosphate dehydrogenase (GAPDH) or Lamin B1 (Cell Signaling Technology, dilution 1:5000) was used as the loading control. After washing, the membranes were incubated with secondary antibody HRP-conjugated goat anti-rabbit (Cell Signalling Technology,dilution 1:5000) for 1h at room temperature and visualised by enhanced chemiluminescence detection kit (Millipore). All experiments were performed in triplicate.

### Co-Immunoprecipitation and immunoblotting analysis

For co-immunoprecipitation analysis, cells lysates were incubated with 5ug antibody on a rotator overnight at 4^o^C. The protein-antibody-protein A/G agarose complexes were prepared by adding protein A/G agarose beads (Invitrogen) for an hour at 4^o^C. After extensive washing with Radio-Immunoprecipitation Assay (RIPA) lysis buffer, the immunoprecipitated complexes were resuspended in reducing sample buffer and boiled for 10 minutes. After centrifugation to pellet the agarose beads, supernatants were subjected to SDS-PAGE and immunoblotting.

### Chromatin immunoprecipitation assay

Chromatin immunoprecipitation (ChIP) was done using the ChIP kit (Millipore) according to the manufacturer's instruction. Briefly, 1×10^7^ HCT116 cells in a 10cm culture dish were treated with 1% formaldehyde to cross-link proteins to DNA. The cell lysates were sonicated to shear DNA to sizes of 100 to 1500 bp. Equal aliquots of chromatin supernatants, 5μg anti-HDGF antibody (Abcam) or anti-IgG as negative control was added, were incubated overnight at 4°C with rocking. After reverse cross-link of protein/DNA complexes to free DNA, real time-PCR was done using specific primers of β-catenin predicted by JASPAR database: forward: 5′-CAA TAG GCA TAT TTA CTA AAC AGG-3′; reverse; 5′-AAC ATA ATA GCA ACA GCT GCA GCC-3′; GAPDH: forward: 5′-GGA GTC CAC TGG CGT CTT-3′; reverse; 5′-CTT GAG GCT GTT GTC ATA CTT C-3′; GAPDH was used as the loading control.

### Luciferase reporter assay

1×10^4^ HCT116 cells were seeded in triplicates in 96-well plates. 0.45ug luciferase reporter plasmid containing β-catenin wild promoter (-648∼-640bp: AGACACAGT; -157∼-150bp: AAGAAATT; -54∼-47bp: CAAAGATG) or β-catenin mutant promoter (-648∼-640bp: GTACAGCAA; -157∼-150bp: ATATAGAA; -54∼-47: AGCAATAG) and 0.45ug pRL-TK Renilla plasmid (Promega) were transfected into cells using the Lipofectamine 2000 reagent (Invitrogen). After 48 hours transfection, Luciferase and Renilla signals were measured using the Dual-Luciferase Reporter Assay kit (Promega). Three independent experiments were done and the data were presented as the mean±SD.

### Immunohistochemistry and evaluation

As described previously [8], the working concentrations of primary antibody for the detection of HDGF (Sigma, St. Louis, MO) and β-catenin (Cell Signaling Technology, Danvers, MA) were 1: 50 and 1:100, respectively. The degree of HDGF immunostaining was defined as the proportion score multiplied by the staining intensity score. The percentage of nuclear HDGF-labeled tumor cells was calculated among at least 1,000 carcinoma cells. Staining intensity was graded according to the following criteria: 0 (no staining); 1 (weak staining = light yellow), 2 (moderate staining = yellow brown), and 3 (strong staining = brown). Each tissue section was calculated in 5 different areas and an average score was used for subsequent analyses. The final immunostaining score of each tumor was the average of scores generated by the two observers.

The index of nuclear HDGF expression was further divided into a high-HDGF expression group and low-HDGF expression group according to the cut-off point that was the mean staining score of all cases. The staining of β-catenin was scored according to Maruyama's method [25]. When more than 70% of carcinoma cells were positively stained for membranous β-catenin, the cells was classified as β-catenin normal expression; if more than 10% of carcinoma cells were positively stained for cytoplasm or nuclei was regarded as β-catenin abnormal expression.

### Xenograft tumor model

Female BALB/c-nude mice (4-5 weeks old and weighing 15-18g) were housed under pathogen-free conditions. HT29 cells were trypsinized, washed twice with serum-free medium and reconstituted in serum-free medium DMEM, mixed 1:1 with Matrigel (Becton-Dickinson) and then inoculated subcutaneously into the right flank of each nude mouse. A local HDGF siRNA treatment was initiated when the tumor was palpable at a volume of approximately 50 mm^3^. The mice were randomly assigned into treatment and negative control groups (*n* = 6 mice/group) and given intratumour injection with 2nM HDGF siRNA or NC-siRNA dissolved in 30μl PBS every 3 days. We modified siRNA with 2-O-methyl and conjugated cholesterol to the ends of the siRNA, which can retain full potency of the siRNA, confers substantial nuclease resistance, improves bio-distribution and facilitates entry into cells [26]. The treatment time was 12 days. Tumor size was measures every 3 days, using a digital caliper, and the tumor volume was calculated according to the formula: tumor volume (mm^3^) = length×width^2^×0.5. At the end of the experiment, all mice were sacrificed and the total weights, tumor weights, and the tumor volumes were recorded. All the experiments were performed following the Guide for the Care and Use of Laboratory Animals (National Institutes of Health publication).

### Statistical analyses

Chi-square test and paired *t* test were used to compare the levels of HDGF and β-catenin expression with different groups and various clinicopathological parameters. The Kaplan-Meier survival curves were used to estimate OS. The significance of predictor variables for OS was evaluated by the long-rank test. Prognostic factors associated with OS were investigated according to the Cox proportional hazards regression model in a stepwise manner. Only those factors that were statistically significant (p < 0.05) in the univariate survival analysis were included in the multivariate analyses. Groups from cell culture and *in vivo* experiments were compared using an unpaired, two-tailed Student's tests and results were presented as mean ± SD. For MTS assay, comparison was done by univariate variance analysis (two-way ANOVA). Statistical analyses were performed using SPSS 16.0 statistical software. P < 0.05 was considered to be statistically significant.

## References

[1] Nakamura H, Kambe H, Egawa T, Kimura Y, Ito H, Hayashi E, Yamamoto H, Sato J, Kishimoto S (1989). Partial purification and characterization of human hepatoma-derived growth factor. CLIN CHIM ACTA.

[2] Yamamoto S, Tomita Y, Hoshida Y, Morii E, Yasuda T, Doki Y, Aozasa K, Uyama H, Nakamura H, Monden M (2007). Expression level of hepatoma-derived growth factor correlates with tumor recurrence of esophageal carcinoma. ANN SURG ONCOL.

[3] Yoshida K, Tomita Y, Okuda Y, Yamamoto S, Enomoto H, Uyama H, Ito H, Hoshida Y, Aozasa K, Nagano H, Sakon M, Kawase I, Monden M, Nakamura H (2006). Hepatoma-derived growth factor is a novel prognostic factor for hepatocellular carcinoma. ANN SURG ONCOL.

[4] Iwasaki T, Nakagawa K, Nakamura H, Takada Y, Matsui K, Kawahara K (2005). Hepatoma-derived growth factor as a prognostic marker in completely resected non-small-cell lung cancer. ONCOL REP.

[5] Guo S, Liu HD, Liu YF, Liu L, Sun Q, Cui XJ (2015). Hepatoma-derived growth factor: a novel prognostic biomarker in intrahepatic cholangiocarcinoma. TUMOUR BIOL.

[6] Tsai CC, Huang SC, Tai MH, Chien CC, Huang CC, Hsu YC (2014). Hepatoma-derived growth factor upregulation is correlated with prognostic factors of early-stage cervical adenocarcinoma. INT J MOL SCI.

[7] Tao F, Ye MF, Sun AJ, Lv JQ, Xu GG, Jing YM, Wang W (2014). Prognostic significance of nuclear hepatoma-derived growth factor expression in gallbladder cancer. World J Gastroenterol.

[8] Yang Y, Li H, Zhang F, Shi H, Zhen T, Dai S, Kang L, Liang Y, Wang J, Han A (2013). Clinical and biological significance of hepatoma-derived growth factor in Ewing's sarcoma. J PATHOL.

[9] Lepourcelet M, Tou L, Cai L, Sawada J, Lazar AJ, Glickman JN, Williamson JA, Everett AD, Redston M, Fox EA, Nakatani Y, Shivdasani RA (2005). Insights into developmental mechanisms and cancers in the mammalian intestine derived from serial analysis of gene expression and study of the hepatoma-derived growth factor (HDGF). DEVELOPMENT.

[10] Liao F, Dong W, Fan L (2010). Apoptosis of human colorectal carcinoma cells is induced by blocking hepatoma-derived growth factor. MED ONCOL.

[11] Wong NA, Pignatelli M (2002). Beta-catenin--a linchpin in colorectal carcinogenesis?. AM J PATHOL.

[12] Zhen T, Dai S, Li H, Yang Y, Kang L, Shi H, Zhang F, Yang D, Cai S, He Y, Liang Y, Han A (2014). MACC1 promotes carcinogenesis of colorectal cancer via beta-catenin signaling pathway. ONCOTARGET.

[13] Zhang F, Yang Q, Meng F, Shi H, Li H, Liang Y, Han A (2013). Astrocyte elevated gene-1 interacts with beta-catenin and increases migration and invasion of colorectal carcinoma. MOL CARCINOG.

[14] Zhang QQ, Zhou DL, Lei Y, Zheng L, Chen SX, Gou HJ, Gu QL, He XD, Lan T, Qi CL, Li JC, Ding YQ, Qiao L, Wang LJ (2015). Slit2/Robo1 signaling promotes intestinal tumorigenesis through Src-mediated activation of the Wnt/beta-catenin pathway. ONCOTARGET.

[15] Yoo BH, Masson O, Li Y, Khan IA, Gowda PS, Rosen KV (2014). Anoikis of colon carcinoma cells triggered by beta-catenin loss can be enhanced by tumor necrosis factor receptor 1 antagonists. ONCOGENE.

[16] Chen X, Yun J, Fei F, Yi J, Tian R, Li S, Gan X (2012). Prognostic value of nuclear hepatoma-derived growth factor (HDGF) localization in patients with breast cancer. PATHOL RES PRACT.

[17] Yamamoto S, Tomita Y, Hoshida Y, Takiguchi S, Fujiwara Y, Yasuda T, Doki Y, Yoshida K, Aozasa K, Nakamura H, Monden M (2006). Expression of hepatoma-derived growth factor is correlated with lymph node metastasis and prognosis of gastric carcinoma. CLIN CANCER RES.

[18] Ren H, Chu Z, Mao L (2009). Antibodies targeting hepatoma-derived growth factor as a novel strategy in treating lung cancer. MOL CANCER THER.

[19] Liao F, Liu M, Lv L, Dong W (2010). Hepatoma-derived growth factor promotes the resistance to anti-tumor effects of nordihydroguaiaretic acid in colorectal cancer cells. EUR J PHARMACOL.

[20] Mao J, Xu Z, Fang Y, Wang H, Xu J, Ye J, Zheng S, Zhu Y (2008). Hepatoma-derived growth factor involved in the carcinogenesis of gastric epithelial cells through promotion of cell proliferation by Erk1/2 activation. CANCER SCI.

[21] Song Y, Hu Z, Long H, Peng Y, Zhang X, Que T, Zheng S, Li Z, Wang G, Yi L, Liu Z, Fang W, Qi S (2014). A complex mechanism for HDGF-mediated cell growth, migration, invasion, and TMZ chemosensitivity in glioma. J NEUROONCOL.

[22] Kang DW, Min DS (2010). Positive feedback regulation between phospholipase D and Wnt signaling promotes Wnt-driven anchorage-independent growth of colorectal cancer cells. PLOS ONE.

[23] Lukasik SM, Cierpicki T, Borloz M, Grembecka J, Everett A, Bushweller JH (2006). High resolution structure of the HDGF PWWP domain: a potential DNA binding domain. PROTEIN SCI.

[24] Yang J, Everett AD (2007). Hepatoma-derived growth factor binds DNA through the N-terminal PWWP domain. BMC MOL BIOL.

[25] Maruyama K, Ochiai A, Akimoto S, Nakamura S, Baba S, Moriya Y, Hirohashi S (2000). Cytoplasmic beta-catenin accumulation as a predictor of hematogenous metastasis in human colorectal cancer. ONCOLOGY-BASEL.

[26] Czauderna F, Fechtner M, Dames S, Aygun H, Klippel A, Pronk GJ, Giese K, Kaufmann J (2003). Structural variations and stabilising modifications of synthetic siRNAs in mammalian cells. NUCLEIC ACIDS RES.

